# Pyrimethamine Inhibits Human Ovarian Cancer by Triggering Lethal Mitophagy via Activating the p38/JNK/ERK Pathway

**DOI:** 10.32604/or.2025.063724

**Published:** 2025-08-28

**Authors:** Lingjuan Linghu, Hongying Zhou, Gang Zheng, Tao Yi

**Affiliations:** 1 Development and Related Diseases of Women and Children Key Laboratory of Sichuan Province, Key Laboratory of Birth Defects and Related Diseases of Women and Children, Ministry of Education, West China Second Hospital, Sichuan University, Chengdu, 610041, China; 2 Department of Human Anatomy, West China School of Preclinical and Forensic Medicine, Sichuan University, Chengdu, 610041, China

**Keywords:** Pyrimethamine, drug repurposing, ovarian cancer, mitophagy, P38/JNK/ERK pathway

## Abstract

**Objectives:**

Ovarian cancer, a leading cause of gynecological malignancy-related mortality, is characterized by limited therapeutic options and a poor prognosis. Although pyrimethamine has emerged as a promising candidate demonstrating efficacy in treating various tumors, the precise mechanisms of its antitumor effects remain obscure. This study was specifically designed to investigate the mode of action underlying the antitumor effects of pyrimethamine in preclinical settings.

**Methods:**

The effects of pyrimethamine on cellular proliferation were meticulously assessed using both the cell counting kit 8 (CCK-8) assay and the colony formation assay, with the effects further confirmed in a murine model. A confocal microscope was utilized to monitor the dynamic alterations in mitochondria within ovarian cancer cells. Additionally, adenosine triphosphate (ATP) and reactive oxygen species (ROS) assays were conducted to measure mitochondrial damage induced by pyrimethamine in ovarian cancer cell lines. The mitochondrial membrane potential was assessed using fluorescent dyes as an indicator of mitochondrial functional status. Furthermore, transcriptome analysis and immunohistochemical techniques were employed to detect the impact of pyrimethamine on ovarian cancer cells.

**Results:**

Our results demonstrated that pyrimethamine induced ovarian cancer cell death through mitochondrial dysfunction and lethal mitophagy. Transcriptome profiling analysis and Western blot demonstrated that activation of the p38/JNK/ERK signaling pathway was implicated in the process of pyrimethamine-induced mitophagy in ovarian cancer cells. Importantly, combination treatment with pyrimethamine and paclitaxel *in vitro* and *in vivo* showed a synergistic antitumor effect.

**Conclusions:**

Altogether, these findings indicate that the antitumor effects of pyrimethamine result from the induction of lethal mitophagy via regulation of the p38/JNK/ERK pathway in ovarian cancer. Considering the low toxicity and high tolerance associated with pyrimethamine, it is suggested that pyrimethamine be evaluated in the treatment of ovarian cancer, either as a monotherapy or in combination with paclitaxel.

## 1 Introduction

Due to the lack of reliable early detection techniques, more than 70% of ovarian cancers are diagnosed at an advanced stage, with the five-year survival rate of approximately 47.4% [1]. International Agency for Research on Cancer (IARC) reported >300,000 ovarian cancer cases and >200,000 deaths globally in 2022. Among them, Asia had 178,223 ovarian cancer patients and 109,547 deaths [2]. Although conventional therapies, such as surgery and chemotherapy, have benefited patients with advanced ovarian cancer, the toxic side effects and drug resistance of chemotherapy seriously affect the treatment effectiveness for ovarian cancer.

Drug repurposing, a drug development strategy, has significant advantages in the medical and oncology fields [3]. Due to their strict drug testing and proven safety in patients, these compounds can significantly reduce the expenses associated with novel drug development. Thus far, successes in oncological drug repurposing have been infrequently reported [4]. Pyrimethamine is an artificially synthesized antimalarial drug inhibiting dihydrofolate reductase of malarial parasites [5]. As a preventative drug in clinical practice, the intermittent use of sulfadoxine-pyrimethamine is commonly advised for malaria prevention in pregnant African women [6]. Pyrimethamine inhibits dihydrofolate reductase, an enzyme essential for folate metabolism, thereby blocking nucleotide synthesis and cell proliferation in rapidly dividing cells. This mechanism has led to its exploratory use in tumor treatment. It has been reported that pyrimethamine can induce apoptosis in acute myeloid leukemia, pituitary adenoma, melanoma, and glioblastoma [7–9], showing effective antitumor activity. Our previous research has demonstrated that pyrimethamine can significantly inhibit cell proliferation and reduce angiogenesis in mouse models of ovarian cancer, and no significant toxic side effects have been found at the treatment dose [10].

Further research has demonstrated that pyrimethamine induces mitochondrial dysfunction in ovarian cancer cells. Building on these findings, our study aimed to explore the mode of action of pyrimethamine in suppressing tumor growth, either alone or combined with paclitaxel, in the context of ovarian cancer treatment, to provide a novel therapeutic strategy for treating this disease.

## 2 Materials and Methods

### 2.1 Reagents

Antibodies: PINK (ab300623), Parkin (ab73015), LC3-II (ab192890), MT-CO2 (ab110258), and LAMP2 (ab25631) from Abcam (Cambridge, MA, USA); Cleaved Caspase-3 (9664), Caspase-3 (9662), and GAPDH (2118) from Cell Signaling Technology (Beverly, MA, USA); LC3 (14600-1-AP), p62 (18,420-1-AP), and ATG5 (10181-2-AP) from Protein Tech Group ( Chicago, IL, USA); and p38 (CY5262), p-p38 (CY8114), ERK1/2 (CY5487), P-ERK1/2 (CY5277), JNK1/2/3 (CY5490), and P-JNK1/2/3 (CY5541) from Abways (Shanghai, China). Secondary antibodies used in this study included Goat Anti-Rabbit IgG H&L conjugated to Alexa Fluor 594 (ab150080, Abcam), Goat Anti-Rabbit IgG H&L conjugated to Alexa Fluor 488 (ab150077, Abcam), Goat Anti-Mouse IgG (AB0122, Abways), and Goat Anti-Rabbit IgG (H+L) DyLight 405 (AB0171, Abways). Chemicals purchased from Selleck (Houston, TX, USA) include hydroxychloroquine (S4430), bafilomycin A1 (S1413), and Mdivi-1 (S7162). Pyrimethamine was used in this study from Sigma-Aldrich Corp. (SML3579, St. Louis, MO, USA).

### 2.2 Cell Culture

Human ovarian cancer cell lines A2780, SKOV3, ES2, and OVCA8 were provided by the State Key Laboratory of Biotherapy (Chengdu, Sichuan, China). The normal ovarian epithelial cell line IOSE80 was kindly provided by Ms. Liu from West China Second University Hospital, Sichuan University. All cells were cultured in high-glucose (4.5 g/L) Dulbecco’s Modified Eagle Medium (DMEM, 11965092, Gibco, CA, USA) with 10% fetal bovine serum (Gibco, 10099141C, USA) and a mixture of antibiotics (100 units/mL penicillin and 100 µg/mL streptomycin) (Gibco, 2585616, USA), at 37°C with 5% CO_2_. Using the MycoAlert mycoplasma detection kit (Lonza, LT07-318, Frederick, MD, USA), regular mycoplasma testing was performed on the cell lines in the study.

#### 2.2.1 Cell Counting Kit-8 (CCK-8) Assay

We determined the cell viability using the CCK-8 assay [11]. Cells were plated into 96-well plates (1 × 10^4^ cells/well) overnight at 37°C with 5% CO_2_. After a 48-h treatment period, the supernatant was removed. Then, 10 μL of CCK-8 reagent (Beyotime, C0048L, Nantong, China) plus 100 μL of serum-free medium to each well, and the plates were incubated for 4 h under standard conditions. To evaluate cell viability, the absorption was measured at 450 nm by CMax Plus (Molecular Devices, San Jose, CA, USA).

#### 2.2.2 Clonogenic Assay

A2780 cells were seeded 600 cells/well in 6-well plates and added with pyrimethamine (20 μM), paclitaxel (100 nM), or a combination of both (pyrimethamine 10 μM + paclitaxel 50 nM) for 10 days. Following two washes with PBS, the cells were fixed in 4% paraformaldehyde for 15 min, stained with 0.1% crystal violet for 20 min, and then imaged and quantified using the ImageJ software package (ImageJ 1.54a, National Institutes of Health, Bethesda, MD, USA).

### 2.3 Western Blot Analysis

Cells were lysed with 1 mm PMSF (Beyotime, ST505, Nantong, China) in Ripa buffer (Beyotime, P0013B, Nantong, China) to obtain total protein, which was determined with the BCA protein assay kit (Beyotime, P0012, Nantong, China). Following separation by 10% SDS-PAGE, proteins were transferred to polyvinylidene fluoride (PVDF) membranes (Millipore Corp., Bedford, MA, USA) and blocked with 5% bovine serum albumin (BSA, CWBIO, CW0054M, Taizhou, China). Primary antibody incubation (1:1000) was performed overnight at 4°C, followed by TTBS washes and 45 min incubation with secondary antibodies (1:2000) at 37°C. The antibody binding was detected via chemiluminescence and the resulting signal was imaged by the ChemiDOC MP System (Bio-Rad, Hercules, CA, USA).

#### 2.3.1 Transmission Electron Microscopy (TEM) Assay

After 12 h pyrimethamine treatment, A2780/SKOV3 cells were fixed in 2.5% glutaraldehyde (0.1 M PBS, pH 7.4, 4°C, 4–6 h), post-fixed with 1% osmium tetroxide for 1 h and rinsed. Samples underwent ethanol/acetone dehydration, epoxy resin embedding at 60°C for 48 h, and ultramicrotome sectioning (60–90 nm). Grid-mounted sections were stained with uranyl acetate/lead citrate and imaged by TEM (80–120 kV, 10,000×) (Hitachi, HT7800, Tokyo, Japan) for mitochondrial analysis.

#### 2.3.2 Immunofluorescence Assay

Cells were fixed in 4% paraformaldehyde (10 min), permeabilized with 0.1% Triton X-100 (10 min), then blocked with 10% goat serum in PBST (phosphate buffer saline with 0.05% Tween-20) (1 h, 37°C). Then the slides were incubated with primary antibodies (dilution 1:1000) at 4°C overnight. After three washes with PBS, the slides were subjected to a 45-min incubation with fluorescently labeled secondary antibodies (dilution 1:500). 4^′^,6-diamidino-2-phenylindole (DAPI, Beyotime, Nantong, China) was used to stained the nuclei for 5 mins. The prepared slides were finally examined and analyzed by a fluorescence confocal microscope (Olympus, FV1000, Japan).

### 2.4 Detection of ATP Levels

ATP Fluorometric Assay Kit (ab83355, Abcam) was utilized to detect the cellular ATP levels by the manufacturer’s instructions [12]. We harvested 1 × 10^6^ cells for each assay, washed the cells with cold PBS, resuspended them, and homogenized them quickly by pipetting them up and down a few times. After centrifugation and supernatant collection, 50 µL of Background Reaction Mix was added to the background wells, and 50 µL of Reaction Mix was added to the standard and sample wells. After mixing and incubating in the dark for 30 min, the fluorescence output was quantified using a spectramax® absorbance reader (Molecular Devices, CA, USA) at Ex/Em = 535/587 nm.

#### 2.4.1 Cellular ROS Detection Assay

In this assay, Cellular ROS Assay (ab113851, Abcam) was used [13]. 2 × 10^4^ SKOV3 or A2780 cells were plated in 6-well plates and incubated with different concentrations of pyrimethamine (0, 20, 40, 80, and 160 μM). After 24 h, collect the cells and inoculate them into a 96-well plate overnight. Subsequently, 10 μM DCFH-DA were added to the cells at 37°C for 45 min, then the cells were collected, subjected to two washes with PBS, and resuspended for subsequent analysis. Finally, we detected the results by spectramax® absorbance reader at Ex/Em = 485/535 nm.

#### 2.4.2 MT-Keima Assay

To detect mitophagy, the mt-Keima-COX8 lentivirus was obtained from Vigen Biotech (LV01230-2a, Zhenjiang, China) [14]. Seed A2780 cells in poly-L-lysine-coated 35 mm dishes and allow them to reach 60–70% confluency before viral infection. To induce mitophagy, treat cells with 20 μM pyrimethamine for 24 h, then wash them with PBS prior to imaging. Images using a confocal microscope with dual-excitation lasers (440 nm and 550 nm, emission: 620 nm) under 60× oil objectives. The green signal (440 nm) reflects mitochondria-localized Keima, while the red signal (550 nm) indicates lysosomal delivery. Quantify the red-to-green fluorescence ratio (mitophagy index = Red/[Red + Green]) using ImageJ.

#### 2.4.3 LAMP2/MT-CO2 Colocalization Assay

To assess mitophagy through LAMP2 (lysosomal marker) and MT-CO2 (mitochondrial COX2 subunit) colocalization analysis, cells at 60–70% confluency were treated with 20 µM pyrimethamine for 24 h to induce mitophagy. Fix cells with 4% paraformaldehyde (15 min), permeabilize with 0.1% Triton X-100 (10 min), and block with 5% BSA 1 h. Incubate with primary antibodies: anti-LAMP2 (1:500) and anti-MT-CO2 (1:500) overnight at 4°C, respectively. After washing, apply secondary antibodies (1:1000) for 1 h at room temperature, followed by DAPI nuclear staining 5 min. Image with a confocal microscope using a 60× oil-immersion objective and analyze colocalization with ImageJ.

#### 2.4.4 Mitochondrial Polarization Assay

The mitochondrial polarization was assessed utilizing JC-10 (ab112134, Abcam), a dye that selectively targets mitochondria and exhibits a reversible color transition from red to green in correlation with the decline in membrane potential [15]. A2780 cells were plated in 6-well plates and incubated with gradient concentrations of pyrimethamine (0, 20, 40, 80, and 160 μM) for 24 h, then collected and inoculated the cells into a 96-well plate overnight. Next, 50 μL/well of 1 × JC-10 dye-loading solution was added in the dark for 30 min at 37°C, then detecting JC-10 monomers by fluorescence microscopy (Olympus FV1000, Japan).

### 2.5 Functional Enrichment Analysis

KEGG pathway enrichment as well as GO analysis were conducted, and the threshold for significantly enriched KEGG pathway was Padj ≤ 0.05 [16]. We use GSEA to determine the enrichment of different gene sets in specific biological processes between pyrimethamine-treated and control groups, considering enrichment scores > 0.45 and *p* < 0.05 as statistically significant.

#### 2.5.1 In Vivo Mouse Model

All animal experiments were approved by the Animal Ethics Committee of West China Second Hospital (2022007-1), ensuring ethical standards were maintained throughout the study. For drug treatment, BALB/c nude mice (20–25 g, 4–6 weeks) were purchased from Vital River Laboratory Animal Technology Co., Ltd. (Beijing, China) were selected and intraperitoneally injected with a total of 2 × 10^6^ A2780 cells to establish an intraperitoneal carcinomatosis model. The mice were randomly divided into four groups (*n* = 8/group): (a) mice injected with 0.9% normal saline (NS); (b) mice injected with pyrimethamine (15 mg/kg); (c) mice injected with paclitaxel (5 mg/kg); and (d) mice injected with pyrimethamine (7.5 mg/kg) + paclitaxel (2.5 mg/kg). Treatment commenced five days after tumor cell injection and was delivered intraperitoneally (i.p.) every week, for a total of four administrations. We monitored the mice’s weight and cachexia every other day. One week after the last dose, half of the mice were euthanized, following which the tumors were surgically removed and measured. The remaining mice were kept to observe the survival rate.

### 2.6 Statistical Analyses

Data are presented as the mean ± standard deviation (SD). Each *in vitro* experimental procedure was replicated at least three times. Statistical analyses were performed using either independent *t*-tests or one-way ANOVA, as appropriate, with Tukey’s post-hoc test for multiple comparisons. A *p* value of <0.05 was considered statistically significant.

## 3 Results

### 3.1 Pyrimethamine Induces Autophagic Cell Death in Ovarian Cancer Cells

**Figure 1:**
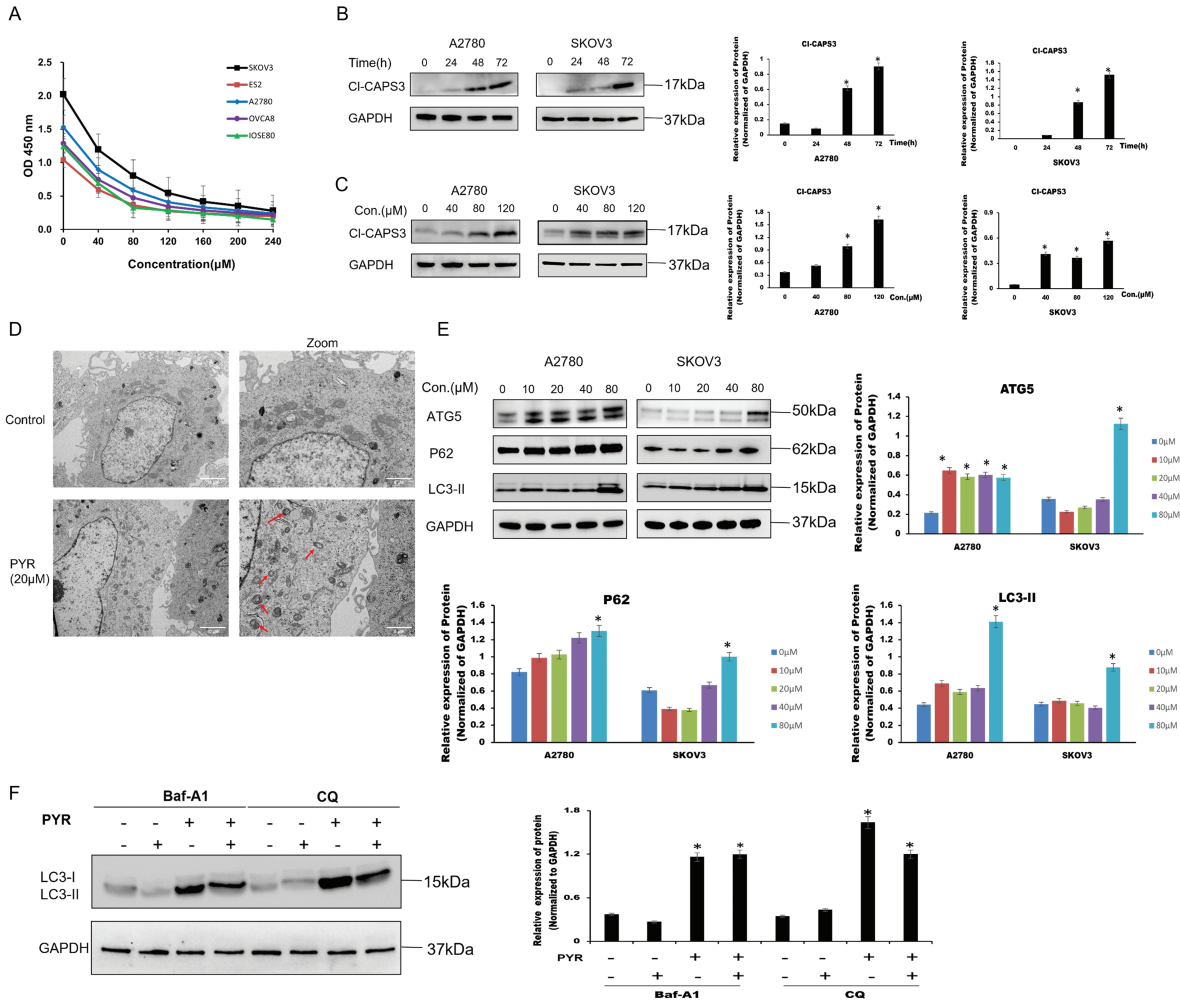
Pyrimethamine induced autophagic cell death in ovarian cancer cells. (**A**) Growth inhibition effect of pyrimethamine (PYR) on A2780s, SKOV3, ES2, OVCAR8, and IOSE80 cells. After 48 h PYR exposure, cell viability was measured by CCK-8. (**B**) Effect of PYR treatment on cleavage Caspase-3 (Cl-CASP3) in ovarian cancer cells. Cells were treated with 20 µM PYR for 0, 24, 48, and 72 h and subjected to western blotting. (**C**) Effect of PYR on Cl-CASP3 expression in ovarian cancer cells. Cells were treated with varying concentrations of PYR for 24 h followed by Western blot analysis. (**D**) Representative images of TEM showing damaged mitochondria (red arrow) after 20 µM PYR treatment for 24 hin SKOV3 cells. Scale bar: 2 μm. (**E**) PYR modulates autophagy-related proteins expression. 24-h PYR treatment at indicated concentrations, cells were followed by western blotting of ATG5, p62, and LC3-II expression. (**F**) Autophagy inhibitors (Baf-A1and CQ) effected PYR-induced LC3 expression. Cells were pretreated with Baf-A1 or CQ for 1 h prior to 20 µM PYR treatment for 24 h, followed by western blotting of LC3, *p < 0.05.

The blocking effect of pyrimethamine on cell growth was evaluated in cell lines SKOV3, A2780, OVCAR8 and ES2, with the regular ovarian epithelial cell line IOSE80 used as a regular control. Pyrimethamine treatment suppressed ovarian cancer cell viability in a dose-dependent manner, with IC50 values ranging between 20 to 60 μM (Fig. 1A). Apoptosis-related biomarkers were first measured. The 24-h administration of pyrimethamine did not trigger caspase-3 cleavage in ovarian cancer cells and the apoptotic signal was only observed at 48 or 72 h (Fig. 1B) or higher pyrimethamine concentrations (Fig. 1C). Our previous study confirmed that apoptosis signal was only measurable at higher pyrimethamine concentrations, and flow cytometry for apoptosis detection *in vitro* yielded the same results. The data showed that apoptosis was not the main cause of cell death.

As transmission electron microscopy (TEM) shown, the number of autophagosomes increased following pyrimethamine treatment (Fig. 1D), and this was accompanied by a dose-dependent elevation in autophagy marker expression (LC3-II, ATG5) in ovarian cancer cells (Fig. 1E). These findings confirm that pyrimethamine activates autophagy in ovarian cancer cells. The autophagy substrate p62 regulates the ubiquitination of chromatin when DNA damage occurs [17]. The levels of LC3-II and p62 can be used as an indirect approach to assess whether autophagosomes are present [18]. After pyrimethamine treatment, a notable elevation in the levels of the p62 protein was detected, indicating a disruption in the ultimate degradation process (Fig. 1E). Bafilomycin A1 (Baf-A1), a classic autophagy inhibitor, was employed to inhibit the fusion process between autophagosomes and lysosomes. Chloroquine (CQ) was used to prevent the degradation phase of autophagic flux by blocking autophagosome fusion and degradation [19]. We found that either Baf-A1 or CQ couldn’t reverse pyrimethamine induced cell death (Fig. 1F). Altogether, these findings demonstrate that pyrimethamine treatment induces autophagy-mediated cell death in ovarian cancer cells.

### 3.2 Pyrimethamine Induces Mitochondrial Dysfunction and Mitophagy in Ovarian Cancer Cells

**Figure 2:**
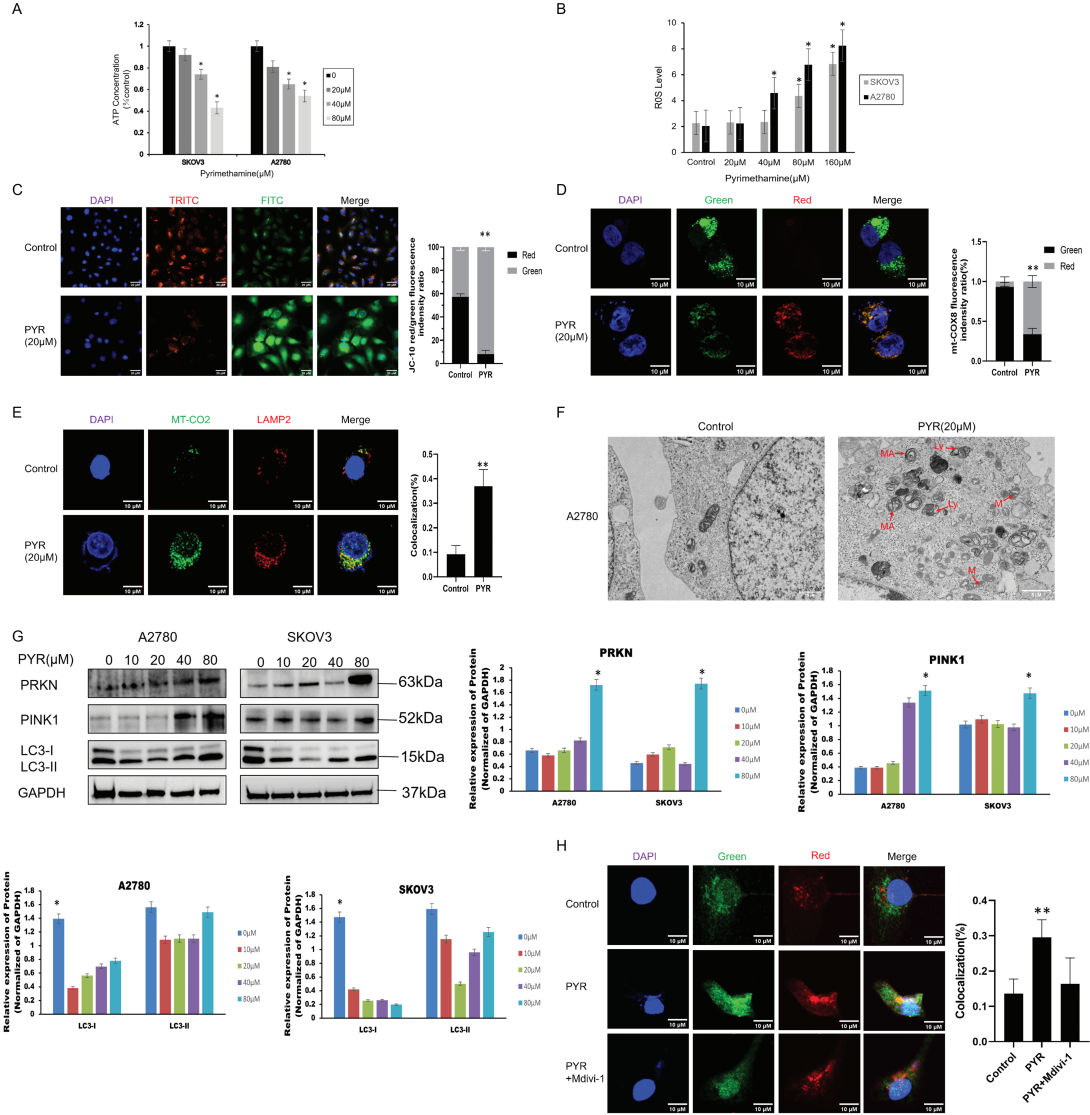
Pyrimethamine induced mitochondrial dysfunction and lethal mitophagy in ovarian cancer cells. (**A**) ATP content was reduced by PYR in a dose-dependent manner. (**B**) ROS content was increased by PYR in a dose-dependent manner. (**C**) JC-10 staining analysis of mitochondrial depolarization in A2780 cells byconfocal microscopy. (**D**) Mitophagy was detected in A2780 cells using the mt-Keima-cox8 assay. The number of mitochondria was detected using confocal microscopy. (**E**) Mitophagy was detected using the LAMP2/MT-CO2 colocalization assay by confocal microscopy. (**F**) Representative TEM images showing the fraction of damaged mitochondria trapped in autophagosomes after 24 h of 20 µM PYR treatment in A2780 cells. MA: mitochondria autophagosome; M: mitochondria; Ly: lysosome, Scale bar: 2 μm. (**G**) Immunoblotting of PRKN, PINK1, and LC3 protein in the mitochondrial fraction in SKOV3 and A2780 cells. (**H**) Effect of Mdivi-1 on PYR treatment, autophagy lysosomes were detected using confocal microscopy (×100). Data are shown as mean ± S.D. and represent three independent experiments, *p < 0.05, **p < 0.01.

We investigated mitochondrial damage caused by pyrimethamine in ovarian cancer cell lines. Decreased ATP levels (Fig. 2A) and increased ROS levels (Fig. 2B) demonstrated that pyrimethamine treatment resulted in mitochondrial damage. JC-10 kit was used to analyze mitochondrial membrane potential in ovarian cancer cells. The transition from red fluorescence to green fluorescence in the cells indicated mitochondrial damage with pyrimethamine treatment (Fig. 2C).

We further explored whether pyrimethamine could induce mitophagy in ovarian cancer. First, the mt-Keima-COX8 was used as a reporter to detect mitophagy signals in ovarian cancer cells [20]. Pyrimethamine stably induced an apparent fluorescence shift from green to red in ovarian cancer cells, indicating that damaged mitochondria are engulfed by autophagic lysosomes (Fig. 2D). We also observed the colocalization between antibodies against mitochondrial (MTCO2) and lysosomal (LAMP2) markers after pyrimethamine treatment (Fig. 2E) [16]. The green-to-red fluorescence shift was typical after pyrimethamine treatment, suggesting a mitophagy pathway. The findings from immunofluorescence assays demonstrated the presence of autophagosomes encapsulating impaired mitochondria within the cells, a phenomenon further validated by transmission electron microscopy (Fig. 2F). Studies indicated that mitophagy and mitochondrial dysfunction may lead to the translocation of PINK1 to the mitochondrial membrane [20]. The PINK1 and PRKN enrichment was observed with the conversion of LC3-I to LC3-II in the mitochondrial fraction of pyrimethamine-treated ovarian cancer cells in a dose-dependent manner (Fig. 2G). Mitochondrial division inhibitor 1 (Mdivi-1), significantly inhibited mitophagy induction and suppressed the toxicity of pyrimethamine in ovarian cancer cells [21], as exhibited by MT-CO2/LAMP2 colocalization (Fig. 2H). The immunofluorescence results showed that autophagosomes in cancer cells contain damaged mitochondria. The findings suggest that pyrimethamine primarily induces mitophagy in ovarian cancer, which is the key mechanism underlying its antitumor effects.

### 3.3 Functional Enrichment Analysis of Pyrimethamine-Induced Mitophagy

**Figure 3:**
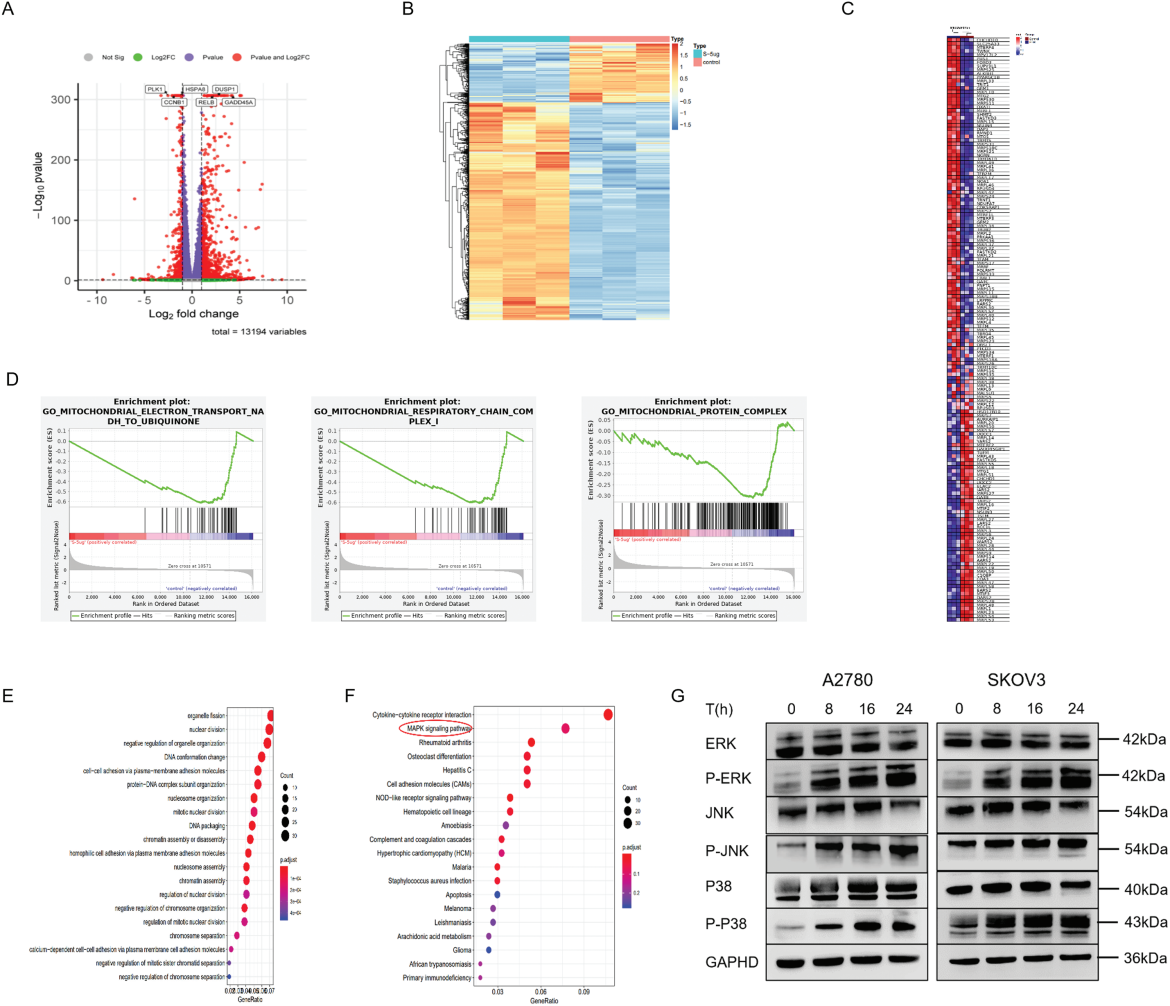
Functional enrichment analysis of PYR-treatment associated gene signatures. (**A**) Transcriptome analysis of PYR on SKOV3 cells. Volcano plot showing significantly differentially expressed genes with upregulated in red and downregulated in green (FDR < 0.05, fold-change > 2). (**B**) Hierarchical clustering is used to perform cluster analysis on gene expression values, and red indicating high expression and blue indicating low expression. (**C**) Heatmap of mitochondrial gene expression between PYR-treatment and control group. (**D**) GSEA enrichment analysis was performed for differentially expressed genes on mitochondrial function. (**E**) The GO analysis for the biological process of differentially expressed genes. (**F**) Dot plot of KEGG pathway enrichment analysis. Dot size represents gene count in each pathway, with color indicating–log_10_(*p*-value). (**G**) PYR regulation of MAPK signaling. Protein levels of total and phosphorylated forms of ERK, JNK, and p38 were analyzed by Western blot following PYR treatment.

To further study the mechanism of pyrimethamine-induced mitochondrial autophagy in ovarian cancer cells, transcriptome analysis was performed on SKOV3 cells. Among 1015 genes with significant differential expression, 217 were downregulated and 798 were upregulated compared with the control group. Specifically, the gene volcano map represents the overall distribution of genes with significant differences between the control and treatment groups, and the expression of NFκB2, RELB, HSPA8, GADD45A, DUSP1, and JUN was significantly upregulated (Fig. 3A). We used hierarchical clustering to perform cluster analysis on gene expression values. In the heat map, genes or samples with similar expression patterns are grouped (Fig. 3B).

Gene Set Enrichment Analysis (GSEA) was used to reveal the differential gene expression of mitochondrion between the pyrimethamine-control and treatment groups, including CHCHD10, SLC25A33, MTERF4, TWNK, FOXO3, MRPS25, ALKBH1, MRPL33 and MRPL3 (Fig. 3C). GSEA analysis also found that mitochondrial-related functions were significantly reduced in the pyrimethamine treatment group, including mitochondrial electron transport NADH to ubiquinone, mitochondrial protein complex, and mitochondrial respiratory chain complex (Fig. 3D). Enrichment at the bottom of control indicates that the gene set of mitochondrial function is downregulated under the overall trend after pyrimethamine-treatment.

Additionally, gene ontology (GO) revealed that the upregulated differentially expressed genes were significantly enriched in processes related to nuclear division, organelle fission, et al. (Fig. 3E). Kyoto Encyclopedia of Genes and Genomes (KEGG) analysis revealed that the upregulated differentially expressed genes were significantly enriched in the MAPK signaling pathway (Fig. 3F). Western blotting analysis indicated that the phosphorylation of p38, JNK, and ERK, which are marker proteins of the MAPK pathway, was significantly activated following pyrimethamine treatment (Figs. 3G and S1). These hub genes and the MAPK signaling pathway might be involved in the pyrimethamine-treatment-induced mitophagy.

### 3.4 Combined Treatment with Pyrimethamine and Paclitaxel for Ovarian Cancer

**Figure 4:**
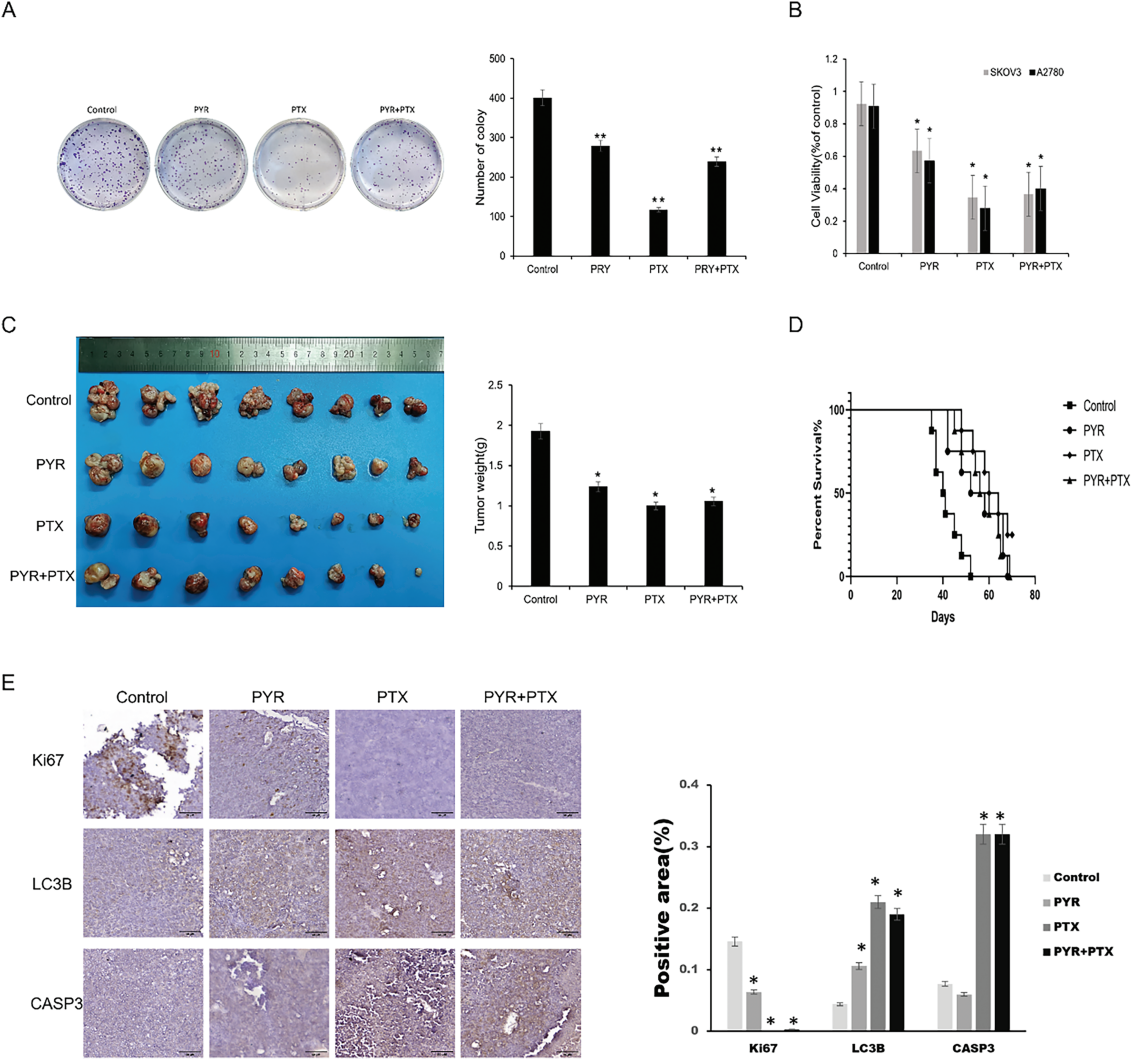
Synergistic effect of pyrimethamine and paclitaxel on ovarian cancer *in vitro* and *in vivo*. (**A**) Combination effect of PYR and paclitaxel (PTX)on the clone formation on SKOV3 cells. SKOV3 cells were treated with PYR (20 μM), PTX (100 nM), or both for 7 days. (**B**) Combination effect of PYR and PTX on the proliferation of ovarian cancer cells. Treated with PYR (20 μM), PTX (100 nM) or both for 48 h, cell viability was measured by CCK-8 assay. (**C**) *In vivo* antitumor activity of PYR in A2780 xenografts. Mice bearing the A2780 tumors were treated with control (*n* = 8), PTX (5 mg/kg, *n* = 8), PYR (15 mg/kg, *n* = 8), or PYR (7.5 mg/kg) and PTX (2.5 mg/kg, *n* = 8) for 30 days. (**D**) Overall survival was assessed by Kaplan-Meier curve analysis. (**E**) Immunohistochemical analysis of proliferation (Ki67), apoptosis (cleaved CASP3), and autophagy (LC3) markers in groups. Quantified data (mean ± SD) demonstrate significant differences between groups *p < 0.05, **p < 0.01.

Paclitaxel is recommended as the primary chemotherapeutic treatment for ovarian cancer, but drug resistance seriously influences its chemotherapeutic effect. Thus, it was expected that pyrimethamine combination with paclitaxel could enhance the clinical efficacy of paclitaxel. The antitumor effects of pyrimethamine, paclitaxel, and pyrimethamine-paclitaxel combination were examined *in vitro*. Compared with the control group, the pyrimethamine–paclitaxel combination enhanced the antitumor effect (Fig. 4A,B). Then mice were treated with pyrimethamine (15 mg/kg) paclitaxel (5 mg/kg) or pyrimethamine (7.5 mg/kg) + paclitaxel (2.5 mg/kg) for 4 weeks. The tumor volume in the combination group was markedly smaller than that of the control group, while the survival rate of mice with tumors in the combination group was significantly higher than that of the control group (*p* < 0.05) (Fig. 4C,D). In Immunohistochemical staining, increased levels of Cl-CASP3 and LC3-II with reduced expression of Ki67 were found in the combination group (Fig. 4E). These results collectively demonstrate that the combination of pyrimethamine and paclitaxel effectively suppresses ovarian cancer cell growth and prolongs survival compared to other treatment groups. This suggests that pyrimethamine can enhance the antitumor efficacy of paclitaxel in the context of ovarian cancer.

## 4 Discussion

Although the inhibitory effect of pyrimethamine on the growth of several cancer cells has been reported, its mechanism is still unclear. Our results indicate that pyrimethamine suppresses the proliferation of ovarian cancer cells *in vitro*. Caspase-3 serves as the primary terminal cleaving enzyme during the process of cell apoptosis, and it is only cleaved during cell apoptosis. Once cleaved, caspase-3 translocated into the nucleus, where it cleaves specific substrates [22]. In-depth studies showed that pyrimethamine treatment of ovarian cancer cells for 24 h did not induce caspase-3 cleavage, and cleaved caspase-3 could only be measured after 48 or 72 h of pyrimethamine administration. These findings indicate that apoptosis is not the primary mechanism of cell death.

Pyrimethamine treatment induced the expression of autophagic biomarkers (LC3-II, ATG5) in ovarian cancer cells in a dose-dependent manner. Through transmission electron microscopy imaging, an increase in autophagosomes was also observed in the cells treated with pyrimethamine, confirming that pyrimethamine induces autophagy in ovarian cancer cells.

Mitochondria are the energy metabolism centers of cells, participating in various life activities and playing essential roles [23]. We found that pyrimethamine caused mitochondrial dysfunction in ovarian cancer cells, accompanied by abnormal changes in mitochondrial ATP, membrane potential, and morphology, ultimately leading to the occurrence of mitochondrial autophagy. Subsequently, we assessed the impact of pyrimidine-induced mitochondrial autophagy on ovarian cancer cells, determining whether it exerts a protective or lethal effect, by employing selective autophagy inhibitors. Cell death induced by pyrimethamine was not reversed by autophagy inhibitors (CQ, Baf-A1). In this study, the Parkin protein and LC3-II/LC3-I ratio increased, indicating that mitochondrial autophagy in ovarian cancer cells was continuously activated after treatment with pyrimethamine, and autophagy levels gradually increased over time. P62 protein is an important selective autophagy junction protein [24], located on the outer membrane of mitochondria. It enters the autophagy lysosomal system through the p62/LC3 pathway and is degraded, thereby clearing protein polymers and damaged organelles [25] to cause mitochondrial autophagy. After administration of pyrimethamine, there was a notable rise in the total protein level of p62, suggesting a disruption in the terminal phase of mitochondrial autophagy, which may lead to pyrimethamine-induced cell death. These findings indicate that ovarian cancer cells initiate mitophagy under pyrimethamine treatment, which culminates in lethal mitophagy.

To further understand the antitumor mechanisms of pyrimethamine treatment on ovarian cancer cells, transcriptomics analysis was adopted, and the gene expression patterns were analyzed comprehensively. In this context, certain genes highlighted in the scatterplot were further identified, and their associations were explored through clustering analysis. Furthermore, GSEA was conducted to examine the impact of pyrimethamine treatment, revealing a downregulation in genes associated with mitochondrial functions. The enrichment results from the KEGG analysis suggest that the MAPK signaling pathway is a critical regulator in pyrimethamine-mediated effects on cellular processes, including growth, migration, proliferation, and apoptosis [26]. The MAPK pathway is crucial in various cellular processes, including cell differentiation, apoptosis, and tumor metastasis. JNK and p38 MAPK are predominantly implicated in responses to cellular stress and apoptosis, whereas ERK is primarily linked to the regulation of cell proliferation and differentiation [27]. The elevated expression levels of p-ERK, p-JNK, and p-p38 have potential anticancer effects in some cases, resulting in MAPK pathway–mediated apoptosis [28–30]. In a previous study, abnormal mitochondrial morphology and functional impairment were also observed linked to ERK signaling pathway alterations in Parkinsonian models [31]. HTR1B regulates mitochondrial homeostasis and mitophagy also by activating the ERK signaling pathway [32]. In this study, we investigated the role of the MAPK pathway in initiating mitophagy by employing Western blot analysis. We observed that the treatment of ovarian cancer cells with pyrimethamine led to a dose-dependent increase in the levels of p-ERK, p-JNK, and p-p38. Taken together, these findings indicate that pyrimethamine induces lethal mitophagy in ovarian cancer cells by activating the MAPK pathway by increasing the expression of p-ERK, p-JNK, and p-p38.

One of the most exciting discoveries in our research is the synergistic therapeutic potential of pyrimethamine in combination with paclitaxel for the treatment of ovarian cancer, as demonstrated in vivo. Administration of the pyrimethamine and paclitaxel combination therapy led to a significant decrease in tumor volume and a considerable prolongation of survival in tumor-bearing mice, in contrast to the efficacy observed when either compound was administered separately. More importantly, compared with paclitaxel alone, the mice in the combination group had fewer side effects and better physical and mental states. Mechanistically speaking, pyrimethamine induces the initiation of mitophagy in ovarian cancer cells, while paclitaxel inhibits cancer cell proliferation by directly acting on the cell division process. Therefore, combining the two is necessary for the combined antitumor effect of paclitaxel. These findings suggest that inducing mitochondrial autophagy and inhibiting tumor cell proliferation may be effective strategies for the treatment of ovarian cancer.

## 5 Conclusion

In summary, pyrimethamine exhibits significant anticancer activity in laboratory studies by inhibiting tumor cell proliferation, inducing lethal mitophagy, and enhancing sensitivity to chemotherapy drugs. It shows broad application prospects in tumor treatment. However, its clinical translation still requires further research to overcome toxicity and drug resistance issues, thereby providing a novel therapeutic strategy for cancer treatment.

## Supplementary Materials

Figure S1 **PYR regulation of MAPK signaling.** Protein levels of total and phosphorylated forms of ERK, JNK, and p38 were analyzed by Western blot following PYR treatment. *, *p* < 0.05.

## Data Availability

The authors confirm that the data supporting the findings of this study are available within the article.

## Supplementary Materials

The supplementary material is available online at https://www.techscience.com/doi/10.32604/or.2025.063724/s1.
