# Supplementary figures and images for: Pyrimethamine Inhibits Human Ovarian Cancer by Triggering Lethal Mitophagy via Activating the p38/JNK/ERK Pathway
Source: Oncol Res. 2025 Aug 28;33(9):2421–34. doi: 10.32604/or.2025.063724 (PMC12408866; doi:10.32604/or.2025.063724)

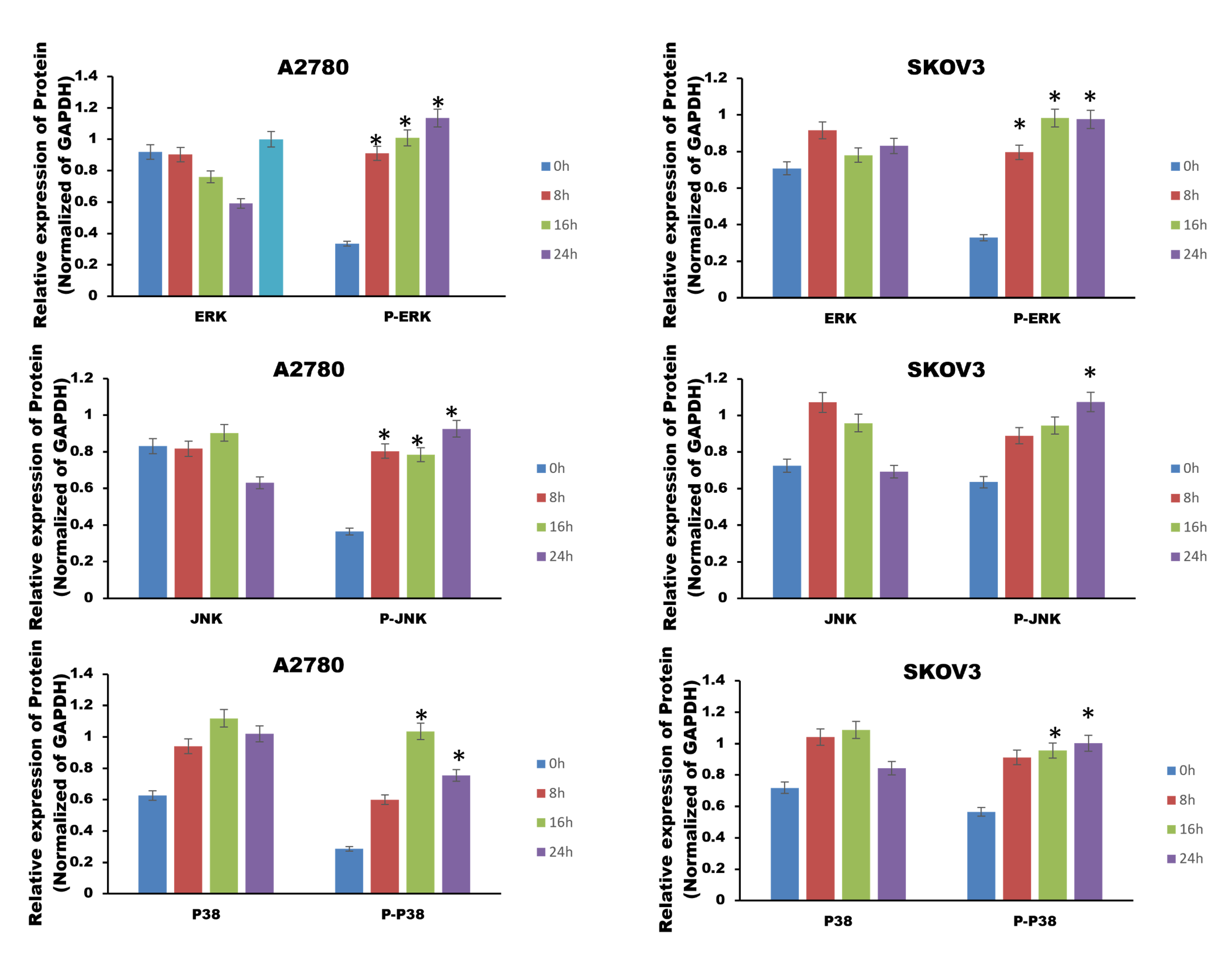

Supplement: Figure S1 [file OncolRes-33-63724-s001.tif]
